# Saccharum

**Published:** 1889-08

**Authors:** E. W. Berridge


### SACCHARUM.

E. W. Berridge, M. D.

In 1881 I met a gentleman who said he was always poisoned by sugar. At my request he wrote the following account: "The first effect on me when I take sugar is to have the tongue furred, and a dry, bitter taste at root of tongue; followed either by sore throat, or running at the nose, as if I had caught a severe cold. With these symptoms is extreme costiveness, lasting at times for two, three, and five days at a time. All these symptoms I can check at once by leaving off sugar. I have at times cured the cold in the head in twenty minutes by drinking copiously of hot water, not less than three pints at a time, sometimes more." On May 28th I gave him, for an experiment, one dose of *Saccharum album*$^{30m}$ (Fincke). On June 16th he reported no change. He was not a patient of mine, and I never heard from him again.

